# Spiritual leadership influence on employee creative service performance: a moderated mediation analysis

**DOI:** 10.1186/s40359-023-01294-0

**Published:** 2023-09-04

**Authors:** Yu-Wen Chiu, Muhammad Waqas Amin, Sheng Tun Li, Muhammad Ali

**Affiliations:** 1 Department of Industrial and Information Management & Institute of Information, Management College of Management, National Cheng Kung University, Tainan City, Taiwan, ROC; 2 School of Management, Yanshan University, Qinhuangdao, China; 3 Department of Industrial and Information Management, National Cheng Kung University, Tainan City, 701 Taiwan, ROC; 4 Federal Urdu University of Arts, Science and Technology, Islamabad, Pakistan

**Keywords:** Spiritual leadership (SPL), Employee autonomy, Proactive personality, Employee creative service performance

## Abstract

This paper expands the understanding of the relationship between spiritual leadership (SPL) and the creative service performance of employees. The research model, based on cognitive evaluation theory, examines the mediating role of employee autonomy and the moderating role of proactive personality in the relationship between SPL and employee creative service performance. Data was collected from 351 employees in China to test the moderated mediation model of this study. The empirical analysis reveals a positive association between SPL and employee autonomy, which in turn leads to increased employee creative service performance. Furthermore, the results show that SPL indirectly affects employee creative service performance via employee autonomy. Additionally, the findings suggest that a proactive personality can enhance the direct effect of SPL on employee autonomy and the indirect effect of SPL on employee creative service performance via employee autonomy. These results contribute significantly to the literature on SPL and creativity. The contributions and implications of this study are discussed in the subsequent section.

**Supplementary Information:**

The online version contains supplementary material available at 10.1186/s40359-023-01294-0.

## Introduction

In information technology (IT) sector is marked by highly dynamic and fierce global competition, relying on scripted ways to serve customers is no longer sufficient. Instead, IT organizations rely on frontline service employees creative service performance to delight customers and solve problems in unique ways [1]. Creative service performance refers to novel and useful ways target at satisfying customer needs [2]. Research has suggested that adopting creative service behavior is critical for attracting and retaining customers in IT industry [3]. Indeed, customer input in IT product development is critical to identify and satisfy customer needs [4]. Moreover, considering the global nature of IT industry, Chinese companies, must complete with other companies around the world to attract and retains customers [5]. Consequently, finding creative ways to satisfy customer needs can provide a competitive advantage to Chinese companies. Thus, frontline service employees, as critical actors in the IT industry, have a privileged position to gather first-hand information on customers’ preferences [6]. This information can be used to incentivize their creative potential. Frontline service employees hold unstructured jobs that often require them to deviate from standard rules and procedures to meet customers’ unique needs [7]. Therefore, creativity of frontline service employees is crucial to provide services that cater to actual customer needs and wants [8, 9].

Extensive research has investigated the factors that contribute to the creativity [10–12] and among them, leaders have been identified as the most significant factor. Therefore, the need for an effective leadership style has increased more than ever before [13], because leadership is considered to be the one furthermost important element that enhances the creative behavior of employees [14]. Existing research primarily focus on top-down leadership styles (i.e., transformational leadership), where leader holds dominant position. Such leadership styles having extensive decision-making power over has been found to be less effective, especially when it comes to the creativity and usefulness ideas [15]. Recognizing the limitations of this approach, our study proposes a bottom-up approach that fosters a sense of purpose, energy, and motivation among employees to drive creativity [16, 17]. Spiritual leadership (SPL) is one such leadership style that can facilitate this approach.

Research indicates the positive role of SPL in enhancing the creativity of employees [18, 19]. Spirituality in the workplace is described as an important dimension of one’s work life, that considered the value of personal feelings and objectives beyond the fulfillment of economic needs [20]. SPL is considered as a behavior of the leader that incorporates three key factors vision of an organization, hope/faith, altruistic love to motivate subordinates, and giving meaning in collective efforts towards achieving the goals [21]. As people working in organizations not only bring in their expertise but they bring their whole selves to the workplace and the spiritual self is the most important aspect of an individual [22]. This study described five dimensions of the SPL including vision, hope, altruistic love, meaning, and membership. Leadership spirituality can be operationally defined as creating a strong vision of the organization, having hope and faith in the vision, creating meaning in work for the employees with love and affection, and giving the feeling of being a member of the team [23]. The term vision was very rare in management literature till the early 1980s but after that growing competition in the world of business forced corporations to use strong visions for their entities [24]. *Vision* defines who we are and what we do [25]. H*ope* is a desire that the vision or the goal set in the past will be achieved, but when there is an addition of faith to this desire then there becomes a certainty of achievement of goals. This is the faith that the vision of the organization will be achieved [26]. Fry [21] in his study relating to SPL noted that *altruistic love* is the feeling of team ship and concern for each member. An employee gets connected with the work spiritually when he feels a *meaning* to his work and he becomes more committed to the job performance [27]. Someone’s feeling that being a *member* of a group he is understood by others and is appreciated [21].

Model of the intrinsic motivation is the building block of cognitive evaluation theory. Intrinsic motivation denotes an innate inclination to pursue challenges and creativity, to enhance the capacity to learn in a given environment [28]. SPL intends to create an environment that enhances the inner satisfaction of people by creating meaning in their work encourages learning and innovation [17]. This research aims to investigate the impact of SPL at the level of frontline employee creative service performance. Firstly, this study aims at exploring whether SPL encourages autonomy in the workplace among followers. Autonomy in the workplace is fundamental for better performance of the employees in the current environment [29]. Autonomy is said to be the extent to which an employee is given the freedom to make decisions on his own during performing different tasks at the workplace such as planning work and the procedures he might follow to complete that particular task [30]. Moller, Deci [31] defined autonomy as a practice or a complete set of practices that allow employees to be independent in making decisions in performing their set tasks. A positive relationship between employee creative service performance and the extent to which they are autonomous in making decisions is indicated in a study by Khoshnaw and Alavi [32]. Having said that the effectiveness of job autonomy on the creative behavior of the workers is indicated positively in many studies, but the need to examine the role of job autonomy in the relationship between SPL and employee creative service performance is yet to be explored. Hence, examining that whether autonomy of the employees is an effective variable linking SPL and employee creative service performance is of utmost significance.

Further, the personal traits of individuals are an imperative aspect that can widely influence the degree to which a leadership style affects employees [33]. Cognitive evaluation theory [34] suggests that human activities are predicted by the collaboration of environmental factors (i.e., SPL) and personal traits. This theory reveals that individual traits of employees in a work environment influence enormously the individual attitude and behaviors towards their job [34]. Proactive personality is one individual characteristic that has major implications for employees’ behavior and performance [35, 36]. Previous research work shows that a proactive personality possesses characteristics that can positively forecast the innovative abilities of individuals, it means that more proactive employees tend to be more innovative at work, but in contrast, those who are lower in proactive tend to be less innovative [9]. Seibert, Crant [37] also established that the proactive personality of an individual has a strong positive connotation with the creative abilities of that individual. Although research outcomes have shown a positive relationship between proactive personality and creative behavior very limited literature is available on the conditional effects of proactive personality on the relationship between leadership style and employee autonomy and in turn outcome as the service-related creativity of frontline employees. Hence this study proposes to consider the moderating impact of a proactive personality trait with SPL style concerning the employee creative service performance.

Building upon cognitive evaluation theory, this research extends the existing literature on SPL and employee creative service performance by making three significant contributions. Firstly, this study enriches the literature by examining how leadership spiritually influences employee creative service performance. Secondly, it adds to the literature on autonomy by investigating the connection between SPL and employee creative service performance through employee autonomy. Therefore, this research scrutinizes the mechanism by which SPL enhances employee creative service performance. Thirdly, this study delves further by exploring the moderating role of proactive personality, thereby explaining the factors that affect the influence of SPL on employee autonomy.

### Theoretical background and hypothesis development

Deci and Ryan [34] proposed the cognitive evaluation theory to explain the factors that affect individual behavior and outcomes. According to their theory, individuals have a strong desire for control and autonomy in the workplace. Satisfaction with this desire acts as a motivational drive that positively influences individual behavior and outcomes [38]. Research suggests that the leader’s role in granting and legitimizing such autonomy and control to employees is crucial [1, 39]. As such, research on leadership reveals that servant, transformational, and authentic leadership have the potential to foster employee creative service performance through several mechanisms, including the promotion of a positive work environment [40], the support of individual growth and development [25], and the promotion of employee autonomy [41].

Liu, Chen [42] found that employee autonomy had a mediating effect on the relationship between SPL and employee creative performance, such that SPL was positively related to employee creative performance through the promotion of employee autonomy. The authors also found that proactive personality moderated the indirect effect of SPL on employee creative service performance through employee autonomy, such that the indirect effect was stronger for individuals with a higher level of proactive personality but not for those with a lower level of proactive personality. Santos, Uitdewilligen [43] also found a positive relationship between SPL and employee creative performance, and that this relationship was mediated by creative work involvement, or the extent to which employees are engaged in and committed to their work. Research has suggested that individual differences in values may play a moderating role in the relationship between SPL and employee creative service performance, with individuals who have higher levels of self-transcendence values being more likely to exhibit creative behaviors in response to SPL [19].

Overall, the research on the relationship between SPL and employee creative service performance suggests that this relationship is complex and multi-faceted, and is influenced by a number of mediating and moderating factors. Therefore, the aim of this study is to examine the complex and multi-faceted nature of this relationship and to identify the mechanisms through which servant leadership may foster employee creative service performance. Particularly, we suggest that SPL is a key non-controlling leadership approach to influence employee creative service performance by motivating employees through granting autonomy within their work roles, based on cognitive evaluation theory. Furthermore, we suggest that the influence of SPL is stronger for employees with high proactive personality.

### Spiritual Leadership and employee autonomy

When an individual gets engaged in behaviors that are according to his own choice and free will, is said to have autonomy in his decisions [44]. The behavior of a leader unswervingly affects the autonomous motivation of his followers [45], and characteristics of some leadership styles allow more autonomy to the followers than the other leadership styles (i.e., participative leadership, ethical leadership, and transformational leadership) [46]. According to Baard, Deci [47], psychological needs satisfaction level is positively related to the leader’s support to his or her subordinates, and it increases the level of autonomous motivation and performance of followers. Similarly, a study by Zhang and Yang [16] suggested that the role of employees’ autonomy is a mediator between SPL and occupational calling. Moreover, Zhang and Yang [16] also noted that SPL style and the innovative behavior of the followers are positively related via mediating role of the employee autonomy factor.

The self-determination theory suggests that the work autonomy behavior among employees is developed when their needs for a relationship, competency, and autonomy are fulfilled [45]. Autonomous motivation is generated when triggered by the fulfillment of the psychological needs of employees through the feeling of being understood and appreciated when the SPL accentuates a common vision, hope, belief, and altruistic love [48]. SPL stresses structuring a good vision for an organization and its people that help create harmony among personal interests and the organizational interests and concentrates on compassion for employees and their spirituality, which reduces stress levels of employees and increases feeling of positive work meaning, consequent to that employees feel more psychological freedom and satisfaction in their interactions [49]. Therefore, the psychological need for work autonomy is satisfied. The spiritual leaders while interacting with their subordinates concentrate on information feedback and attempt to encounter their existent needs, career development is taken care of and the competency needs are contented by providing equal development opportunities [50]. Moreover, spiritual leaders appreciate employees for their achievements, also motivate belief amongst followers to achieve even higher goals, and organizational trust and respect are also well translated to the followers [51]. When the basic psychological needs of workers are met such as relationship, competency, and autonomy by the SPL, which in turn encourages employee autonomy [18], hence this study proposes the hypothesis as follows:

#### Hypothesis *1*

*SPL is positively associated with employee autonomy.*

### Employee autonomy and employee creative service performance

As employee autonomy refers to the extent to which an individual impressions independence, freedom, and free will in shaping schedules and the procedures to perform duty at work [52]. Cai, Parker [53] suggested that employees should be given autonomy at work which helps them find meaning in their jobs and grants them the freedom to set and pursue their own goals. According to Ingvaldsen and Rolfsen [54], employee autonomy is a job characteristic that is not only positively related to the commitment and motivation of employees but also encourages employees to be more creative and pursue novel ideas at work. Various past studies endorsed the positive impact of employee autonomy over the innovative and creative behavior of individuals in a job setting [11, 55, 56]. Job autonomy encourages employees to show positive behavior by being innovative and working on new ideas for their given tasks [57].

According to cognitive evaluation theory [34], individual autonomy fosters the highest level of volitional and high-quality intrinsic motivation. Research indicates that autonomy leads to improved intrinsic task motivation [30]. Similarly, a study conducted in the Indian IT sector by Pattnaik and Sahoo [58] suggested that employees who had greater autonomy in their task performance were more likely to demonstrate creative behaviors in their specific job roles. The extent to which employees have freedom and discretion in carrying out their job tasks is likely to enhance their motivation to go the extra mile and improve their performance. Autonomy also extends employees’ perception that their work is meaningful [29]. In the context of customer service, autonomy enhances employees’ skills, growth, and empowers them to take control of their job responsibilities, thereby motivating them to take initiatives in delivering customer services that effectively meet their needs. Consequently, employee autonomy is expected to improve creative customer service performance. Hence this study emphasizes that employees are more creative when they are given more autonomy at work when compared to a setting where they are constantly given directions and are being controlled. Thus, the following hypothesis is proposed:

#### Hypothesis *2*

*Employee autonomy is positively associated with employee creative service performance.*

### The mediating role of employee autonomy

The literature demonstrates that SPL can influence employee behavior through motivational mechanisms. As such, autonomy has been discussed as an important motivational factor that positively influences the behavior and performance of individual employees and groups [58–61]. This research based on cognitive evaluation theory [34] predicted that SPL is associated with employee autonomy (Hypothesis 1) and employee autonomy is associated with employee creative service performance (Hypothesis 2). Based on the above, it is logical to predict that employee autonomy creates a link between SPL and employee creative service performance. Thus, based on cognitive evaluation theory, this research proposes that SPL creates employee perception of autonomy which motivates their behavior leading to increase employee creative service performance. Hence, we hypothesize as:

#### Hypothesis *3*

*Employee autonomy positively mediates the positive relationship between SPL and employee creative service performance.*

### The moderating role of proactive personality

Till now we proposed that cognitive evaluation theory [34] supports the indirect relationship linking SPL with employee creative service performance via employee autonomy. We further propose that cognitive evaluation theory also suggests that individual characteristics can have a moderating influence. For instance, prior research demonstrates that how leadership influences followers depend on the followers’ personality [15, 36, 62]. A proactive personality is one such personality characteristic that explains how leaders influence followers’ behavior and performance in the workplace [63]. Individuals with proactive personality traits tend to plan while anticipating forthcoming events and their probable outcomes and gathering means for prolific changes [64]. Researchers have suggested that a proactive personality is positively correlated with the creative behaviors of the followers toward the fulfillment of their organizational goals [65, 66]. As is evident from previous research work people with higher proactive behavior are more likely to bring changes to their environment rather than adapting to the control factors [67]. They have an inbuilt ability to recognize and custom opportunities, demonstrate initiative, and stick to it until significant changes are accomplished. Liu, Tangirala [68] suggested that persons who display a high degree of proactive behavior are inclined to show strong autonomy and initiative in performing their tasks. Whereas people with low proactive personality are more passive, do not identify any opportunities, do not have the ability to use them to bring in any changes to their environment, and easily surrender to situational forces [69].

Therefore, having in mind the impact of proactive personality on employee autonomy this study argues that proactive personality will show a moderating impact between the relationship of SPL and employee autonomy. This study anticipated that people with high proactive personality will exhibit a stronger impact of SPL on employee autonomy and the other hand people with low proactive personality will exhibit a less strong impact of SPL on employee autonomy and resulting in employee creative service performance. One reason behind is notion is explained by Chiu, Owens [36]. They argued that impact of leadership on employee is stronger when they encounter followers who show an active behavior. That is, if leader is willing to grant autonomy and follower has active behavior. In such case, leaders has stronger influence on employee perceptions and behavior [15] and is more willing to grant autonomy and control to employees. Accordingly, in context of our study where a spiritual leader has an active approach to granting autonomy to follower, we suggest a high proactive personality of the employee will be more aligned to take benefit of such SPL behavior. Hence, the following hypothesis is proposed:

#### Hypothesis

a: *Proactive personality moderates the positive relationship between SPL and employee autonomy such that the relationship will be stronger when employee proactive personality is high than when it is low.*

### Moderated-mediation effect

**Fig. 1:**
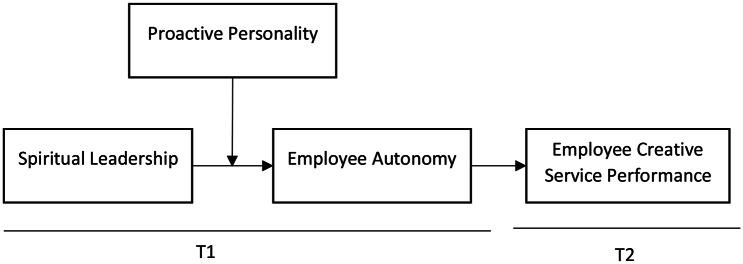
Proposes model

As evident from the depiction of hypothesized relationships in our model (Fig. 1) and according to moderated mediation logic [70, 71], we predict that the influence of SPL towards employee creative service performance via mediating role of employee autonomy can differ due to the moderating role of proactive personality of an individual. Hence, the conditional indirect effect of proactive personality can reinforce the indirect relationship between SPL and employee creative service performance, thus representing a moderated mediation between SPL, employee autonomy, and employee creative service performance. Based upon theoretical discussion and the earlier hypothesis this study antedates that for employees with high proactive personality impact of SPL on employee autonomy and the resulting impact on the employee, employee creative service performance will be stronger. But for employees with weak proactive personality, the impact of SPL on employee autonomy and employee creative service performance will remain weaker. Hence, the following hypothesis is proposed:

#### Hypothesis

b: *The indirect effect of SPL on employee creative service performance via employee autonomy is weakened by the proactive personality, such that the indirect effect of SPL on employee autonomy is stronger when the proactive personality is high than when it is low.*

## Methods

### Sample and procedure

Data was collected from full-time employees working in two information technology organizations in China and their direct supervisors. We identified the organizations using a university-industry network and randomly selected two companies as a sample for testing our study model. We contacted the human resource directors of both companies, briefly explaining the study’s purpose and inviting their organizations to participate in the survey. Upon receiving their approval, we requested the HR department of each organization to assist us contact the employees and their managers who are working together for at least three months. This duration was chosen because this is a reasonable time to development of collective norms among employees and managers.

Data were collected two times from multiple sources including employees and leaders to decrease the potential effect of common method bias [72]. This is a validated approach to collect data [12, 73, 74]. We included a brief purpose of the study, and a statement to inform employees that participate in the survey is voluntary and assured them that their responses will be kept confidential and will not be shared with anyone including the organization. At time one (T1), employees were given a paper-based questionnaire. In this questionnaire, employees rated the spiritual behavior of their leader, employee autonomy, and employee proactive personality, and also provided the details of their demographic characteristics. Employees completed the survey and put the questionnaire in an envelope provided with the questionnaire. The sealed envelope was then returned to the survey team. One month after T1, at time two (T2), questionnaires were provided to direct supervisors of the employees. Leaders were requested to rate the creative performance of their subordinates. Leaders were also provided envelopes with the questionnaires. Leaders put the completed questionnaires in the envelope and returned the sealed envelopes to the survey team members.

At T1, 500 employees were contacted and 367 employees returned the completed questionnaires to the survey team (response rate 0.73%). At T2, supervisors completed and returned 354 questionnaires. After an initial screening, 3 questionnaires were found incomplete by the supervisors resulting in the removal of 3 employee responses. Thus, resultantly, we have 351 complete responses to be used for further analysis. Survey respondents included 202 (57.5%) males and 149 (42.5%) females with 67% age range 21–40 years. In terms of experience, 168 (147.9%) have 1–5 years of experience and 109 (31.1%) have 5–10 years of experience. Among respondents, 78.6% have experience of working with current leader between 7 and 24 months. In terms of education, 178 (50.7%) have at least master degree followed by 157 (44.7%) have bachelor degree. Details of sample characteristics are presented in Table 1.

**Table 1:** Characteristics of the sample

Variable	Category	Frequency	Percentage (%)	Variable	Category	Frequency	Percentage (%)
Gender	Female	149	42.45	Education	High school	16	4.56
	Male	202	57.55		Bachelors	157	44.73
					Masters and above	178	50.71
Age	20 or less	87	24.79	Tenure with leader	6 Months and less	54	15.38
	21–30	115	32.76		7–12 Month	99	28.21
	31–40	120	34.19		13–18 Months	105	29.91
	Above 40	29	8.26		19–24 Months	72	20.51
					25 months and above	21	5.98
Experience	1 year or less	52	14.81	Job type	Marketing & sales	52	14.81
	1–5 years	168	47.86		Accounting and finance	96	27.35
	5–10 years	109	31.05		HRM and administration	84	23.93
	above 10 years	22	6.27		Designing and Production	119	33.90

### Measures

All survey items were taken from existing measures validated in the literature. All the survey measures are included in the supplementary file. Because the context of the study was Chinese employees, therefore, we used a translation and back translation approach to all items in Chinese [75]. All items were rated on a 7 points Likert scale ranging from 1 for strongly disagree to 7 for strongly agree.

#### Spiritual leadership

We used a 9-item scale adapted from Pawar [20] to measure the spiritual behavior of leadership rated by employees at T1 (Cronbach’s alpha of this measure is 0.94). Recent studies have validated this measure [76–78]. A sample item is “My leader carefully listens to subordinates.”

#### Employee autonomy

At T1, employees used 4 items to assess employee autonomy (Cronbach’s alpha of this measure is 0.87). This measure is adopted from Beehr [79]. A sample item is “I have a lot of say over what happens on my job.”

#### Proactive personality

We measured the proactive personality of the employees with 10-item measures developed by Seibert, Crant [37]. Employees rated their proactive personality at T1 (Cronbach’s alpha of this measure is 0.98). A sample item of this measure is “Nothing is more exciting than seeing my ideas turn into reality.”

#### Employee creative service performance

We measured employee creative service performance using 6 items scale adopted from Wang and Netemeyer [80]. The scale is validated by recent study [5]. At T2, direct supervisors of the employees were requested to rate the employee creative service performance (Cronbach’s alpha of this measure is 0.95). Leader ratings are considered a valid approach to measure the creativity of the employees [18, 81–83]. A sample item of this measure is “This employee comes up with new ideas for satisfying customer needs.”

#### Control variables

Literature suggests that the demographic characteristics of the employees can influence their job-related attitude and performance [82, 84, 85]. Accordingly, in order to generate robust results, we controlled employees’ gender, age, education, experience, and tenure with the current leader.

### Analysis and results

We employed a two-step approach commonly utilized in research to test our model. Initially, we utilized AMOS v24 to assess the validity of the data, followed by the use of the PROCESS macro v4.0 to test the hypothesized relationships. These tools are widely regarded as suitable and have been extensively used in previous research to examine moderated mediation models [15, 86, 87].

### Preliminary analyses

In order to assess the discriminant validity of the data, we performed an alternative model test using a set of confirmatory factor analyses using AMOS 24. Results reveal that four factors hypothesized model generates better fit to data (Chi-square = 1283.54, degrees of freedom = 371, root mean square error of approximation = 0.08, the goodness of fit index = 0.79, Tucker-Lewis index = 0.92, comparative fit index = 0.93) than alternative three factors model, two factors model, and one-factor model. The findings of the confirmatory factor analyses are reported in Table 2. The reported results reveal that the measurement model has satisfactory discriminant validity [88].

**Table 2:** Alternative model analysis

Model	Factors	x^2^	df	RMSEA	GFI	TLI	CFA
Model 1	Four factors: Hypothesized model	1283.54	371	0.08	0.79	0.92	0.93
Model 2	Three factors: combined SPL with proactive personality	3348.43	374	0.15	0.44	0.74	0.76
Model 3	Two factors: combined SPL, proactive personality, and employee autonomy	4004.44	376	0.17	0.39	0.69	0.71
Model 4	One factor: combined all variables	8138.98	377	0.24	0.34	0.33	0.38

**Note**: x^2^ = Chi-square; df = degrees of freedom; RMSEA = root mean square error of approximation; GFI = goodness of fit index; TLI = Tucker-Lewis index; CFI = comparative fit index

In this study, we utilized a time-lagged multi-source data approach, wherein the dependent variable was evaluated by the direct supervisors of the respondents. This approach proves beneficial in mitigating potential issues related to common method bias [89]. In addition, research has suggested that moderating relationship cannot be supported if the data is effected by common method bias [90]. In our case, in our case, the significant moderation results provide compelling evidence that our data is not affected by common method bias. Moreover, we applied Harmen’s single-factor analysis [91]. Results reveal that first factor accounted for 38.6% of the variance, and the eigenvalues of the 15 factors exceeded one. These results validate that the findings of this study are not affected by common method bias.

Table 3 shows means, standard deviations, reliability statistics, and correlations among key variables. Significant correlations among key constructs indicate initial evidence of the proposed relationships.

**Table 3:** Correlation, descriptive statistics, and reliability analysis

Variables	Mean	Std. Deviation	1	2	3	4	5	6	7	8	9
1. Gender	0.58	0.49	-								
2. Education	2.46	0.58	-0.06	-							
3. Age range	2.26	0.92	0.00	-0.05	-						
4. Tenure with leader	2.74	1.13	0.01	-0.07	-0.03	-					
5. Experience	2.29	0.79	0.04	-0.10	0.01	-0.02	-				
6. SPL	4.14	0.81	0.01	-0.06	0.03	-0.01	-0.16**	0.94			
7. Employee autonomy	4.32	1.32	-0.05	0.00	0.06	-0.10	-0.05	0.36**	0.87		
8. Proactive personality	3.81	1.33	-0.04	0.01	0.09	-0.03	-0.10	0.36**	0.21**	0.98	
9. Employee creative service performance	3.57	1.19	0.07	0.01	0.01	-0.01	0.00	0.19**	0.25***	0.06	0.95

Note: N = 351, *p ≤ .05, **p ≤ .01, ***p ≤ .001, Cronbach’s alpha in diagonal cells

### Hypotheses analysis

We tested our hypothesized relationships using PROCESS macro v4.0 and reported results in Table 4. The findings provide support for Hypothesis 1, which proposed that SPL is positively related to employee autonomy. As shown in Table 4, SPL is positively related to employee autonomy (β = 0.36, SE = 0.05, p < .001). Hypothesis 2 predicted that employee autonomy is positively related to employee creative service performance. The results (see Table 4) support Hypothesis 2 and that show employee autonomy is positively related to employee creative service performance (β = 0.21, SE = 0.06, p < .001). Hypothesis 3 pretended that employee autonomy positively mediates the positive relationship between SPL and employee creative service performance. As initial evidence of the mediation relationship, Table 4 shows that SPL is positively related to employee autonomy (Hypothesis 1) (β = 0.36, SE = 0.05, p < .001) and employee creative service performance (β = 0.12, SE = 0.06, p < .05), and employee autonomy is positively related to employee creative service performance (β = 0.21, SE = 0.06, p < .001). We used bootstrapping 20,000 replications at 95% confidence intervals to generate confidence intervals to assess the mediation effect of employee autonomy. Results in Table 4 reveal that employee autonomy positively mediates the relationship between SPL and employee creative service performance (β = 0.07, SE = 0.03, [0.03, 0.13]). Thus, findings provide support for the mediation Hypothesis 3.

**Table 4:** Hypothesis analysis

Outcome variable	Employee autonomy			Employee creative service performance			Employee autonomy		
Variable	β	SE	t	β	SE	t	β	SE	t
Constant	0.06	0.35	0.17	-0.26	0.36	-0.72	-0.02	0.34	-0.07
Gender	-0.10	0.10	-1.00	0.14	0.11	1.37	-0.10	0.10	-1.04
Education level	0.03	0.09	0.33	0.03	0.09	0.29	0.05	0.09	0.53
Age range	0.05	0.05	0.99	0.00	0.06	0.05	0.05	0.05	0.90
Tenure with leader	-0.08	0.04	-1.75	0.01	0.05	0.22	-0.06	0.04	-1.43
Experience	0.01	0.06	0.14	0.03	0.07	0.50	0.00	0.06	-0.08
SPL	0.36	0.05	7.07***	0.12	0.06	2.08*	0.36	0.05	6.67***
Employee autonomy				0.21	0.06	3.69***			
Proactive personality							0.13	0.06	2.37*
Interaction							0.13	0.04	2.99**
Mediation	Effect	SE	95%CI -LL	95%CI -UL					
	0.07	0.03	0.03	0.13					

Note: *p ≤ .05; **p ≤ .01; ***p ≤ .001; 95%CI -LL = Lower limit at 95% confidence interval; 95%CI -UL = Upper limit at 95% confidence interval; bootstrapping sample = 20,000; Interaction = SPL x proactive personality; Mediation = Indirect of SPL on employee creative service performance via employee autonomy

#### Hypothesis

**Fig. 2:**
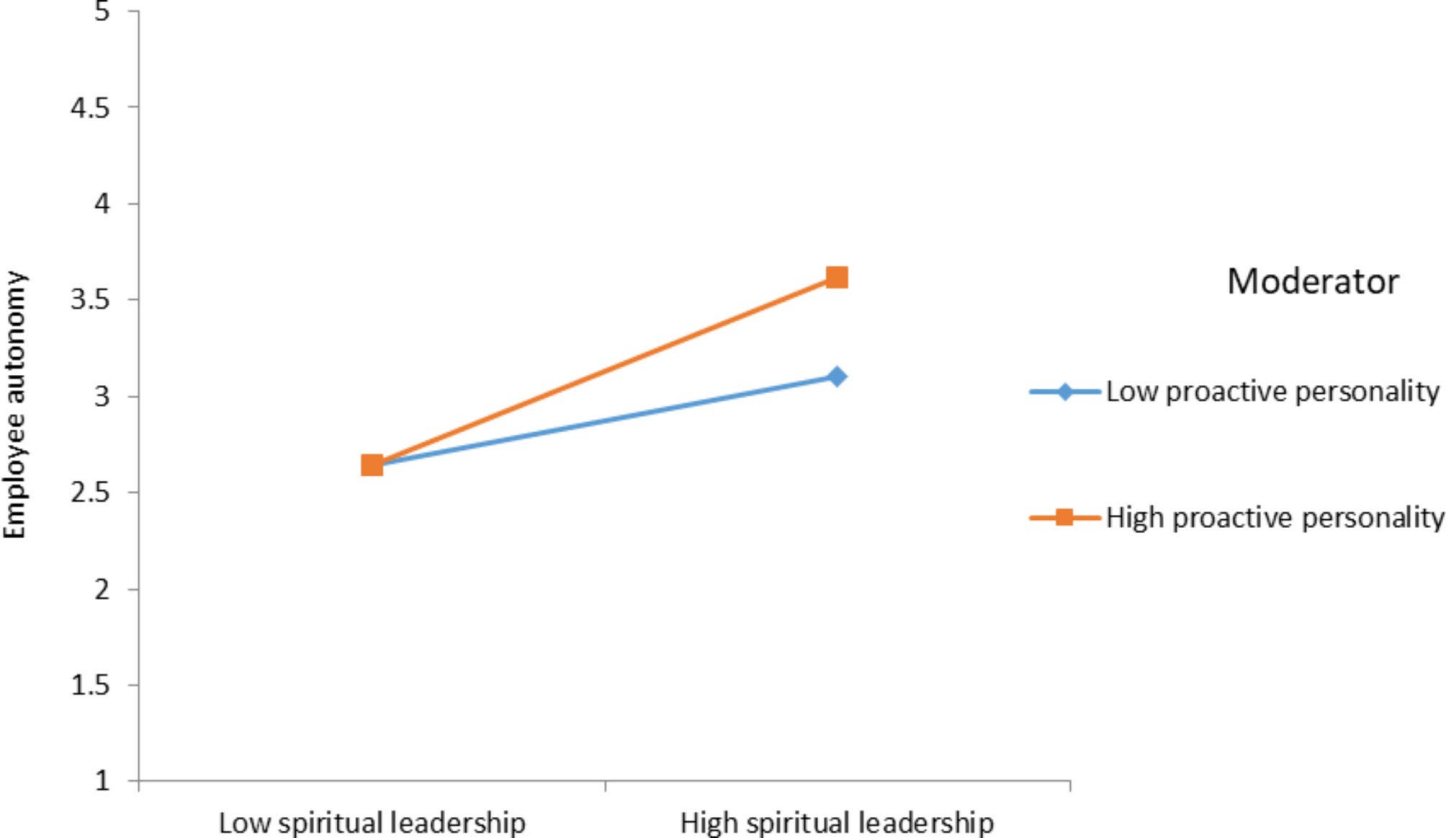
Interaction effect of proactive personality and SPL on employee autonomy

a predicted that proactive personality strengthens the positive relationship between SPL and employee autonomy such that the relationship will be stronger when proactive personality is high than when it is low. Accordingly, results show is a significant interaction effect of proactive personality and SPL on employee autonomy (β = 0.13, SE = 0.04, p < .01). We further validate the moderation effect using a simple slope test. The results of the simple slope test are presented in Fig. 2. Results suggest that SPL was strongly related to employee autonomy at a high level (1 SD above the mean) of proactive personality (β = 0.49, SE = 0.07, p < .001) and was less strongly related to employee autonomy at a low level (1 SD below the mean) of proactive personality (β = 0.23, SE = 0.06, p < .001). These results provide support for Hypothesis 4a.

We further pretended that the indirect effect of SPL on employee creative service performance via employee autonomy is strengthened by the proactive personality, such that the indirect effect of SPL on employee autonomy is stronger when the proactive personality is high than when it is low. Table 5 shows the results for the conditional indirect effects of SPL on employee creative service performance via employee autonomy at levels of proactive personality. Results in Table 5 reveal that the indirect effect of SPL on employee creative service performance via employee autonomy was high at high (1 SD above the mean) of proactive personality (β = 0.10, SE = 0.04, 95% CI = [0.04, 0.18]) than at the mean level of proactive personality (β = 0.08, SE = 0.03, 95% CI = [0.03, 0.13]), and low level (1 SD below the mean) level of proactive personality (β = 0.05, SE = 0.02, 95% CI = [0.02, 0.10]). Furthermore, the index of moderated mediation (index = 0.03, SE = 0.01, 95% CI = [0.01, 0.05]) also provides support for moderated mediation hypothesis. Hence, Hypothesis 4b is supported.

**Table 5:** Conditional indirect effect of SPL on employee creative service performance via employee autonomy at levels of proactive personality

Moderator		Effect	SE	95%CI -LL	95%CI -UL
Proactive personality	(-1SD)	0.05	0.02	0.02	0.10
Proactive personality	Mean	0.08	0.03	0.03	0.13
Proactive personality	(+ 1SD)	0.10	0.04	0.04	0.18

Note: 95%CI -LL = Lower limit at 95% confidence interval; 95%CI -UL = Upper limit at 95% confidence interval; bootstrapping sample = 20,000;

## Discussion and implications

### Discussion

This study aimed to develop and test a moderated mediation model to gain insights into the relationship between employee creative service behavior and SPL. The findings reveal that SPL has a positive influence on granting autonomy to followers. Consequently, employee autonomy positively impacts the creative service performance of frontline service employees in IT companies. The results further indicate that the relationship is moderated by the employee’s proactive personality. Specifically, employees with a proactive personality benefit more from SPL than those with a low proactive personality. Moreover, the indirect effect of SPL on employee creative service performance through employee autonomy is significantly stronger when employee proactive personality is high. These findings suggest that SPL can effectively stimulate creative service behavior through employee autonomy, particularly when employees possess a high level of proactive personality.

### Theoretical contributions

The current study has important theoretical implications. Firstly, prior research has demonstrated that leadership is critical in shaping the service behavior of frontline employees. However, most of these studies have focused on top-down leadership styles that exert strong control over decision-making. As a result, it remains unclear how SPL can influence the creative service performance of frontline employees [16, 20, 92]. This study investigates how SPL can enhance the creative service behavior of frontline employees in the information technology industry. Our findings reveal that SPL has a significant positive effect on employee creative service performance, thus contributing to the literature by establishing a theoretical framework and empirical evidence of the impact of SPL on frontline employees’ creative service performance.

Secondly, previous researchers have suggested the need to study the processes and mechanisms by which SPL may bring creative benefits to employees and organizations [50, 92]. Our study addresses this need by supplementing the available literature on SPL with the perspective of cognitive evaluation theory [34]. We investigate when and how SPL influences employee creative service performance by cultivating employee autonomy. Our results demonstrate that employee autonomy mediates the link between SPL and employee creative service performance. While previous research has found the mediating role of affective commitment, goal orientation, safe relational context, and information exchange in the SPL and creativity association [18, 93], our study shows that SPL fosters a perception of employee autonomy that motivates employees to generate creativity. Employee autonomy is, therefore, an important mechanism underlying the association between SPL and employee creative service performance.

Thirdly, our study contributes to our understanding by investigating the moderating role of individual proactive personality in the association between SPL and employee autonomy. We find that the proactive personality of employees has significant implications for a spiritual leader to grant autonomy to employees. Specifically, our results show that the effect of SPL on employee autonomy is stronger for employees with a higher level of proactive personality than those with a lower level of proactive personality. We, therefore, address the call to investigate moderators that can better explain the influence of SPL on employee behavior and performance [76, 93, 94].

Our results suggest that promoting employee autonomy may be an effective strategy for fostering employee creative service performance, and the effectiveness of this strategy may be enhanced by focusing on individuals with a higher level of proactive personality. This may involve identifying and hiring individuals with high proactive personalities, as well as providing guidance to leaders to demonstrate spirituality, especially with employees with high proactive personalities. Such an approach can significantly increase employee creative service performance and reap the benefits of SPL in organizations.

### Implication for practice

The findings of this study have important practical implications for managers who seek to promote employee creative service performance within their organizations. One key implication is the importance of fostering SPL within the workplace. SPL is characterized by values, attitudes, and behaviors that enable leaders to inspire and motivate others toward the achievement of higher goals and values that transcend self-interest. To foster SPL, managers can create a positive and supportive work environment that encourages employees to think creatively and take risks. This can be achieved by providing opportunities for employees to work on projects that align with their values and interests, promoting work-life balance, and encouraging employees to pursue their passions and interests outside of work.

Another key implication for managers is the importance of promoting employee autonomy. Giving employees greater autonomy in the workplace can lead to higher levels of creativity and innovation, as it allows employees to feel more self-determined and motivated to contribute to the organization. Managers can promote autonomy by providing employees with more control over their work and decision-making processes, as well as offering opportunities for professional development and growth. Encouraging employees to take ownership of their work and be proactive in seeking out new challenges and opportunities can also foster autonomy.

Moreover, managers should consider the moderating role of proactive personality in the relationship between SPL and employee creative service performance. Individuals with a higher level of proactive personality are more likely to exhibit creative behaviors in response to leadership that promotes autonomy and self-direction. This suggests that individuals with a proactive personality may be particularly well-suited to benefit from SPL, which promotes autonomy and employee creative service performance. Managers can foster a proactive personality by providing employees with opportunities to take on leadership roles and initiatives and encouraging them to be proactive in seeking out new challenges and opportunities.

Overall, managers can create a work environment that encourages creativity and innovation by fostering SPL, promoting employee autonomy, and supporting the development of a proactive personality. These practices can lead to enhanced employee creative service behavior of frontline service employees.

### Limitations and future research directions

While our study offers several valuable theoretical implications, it is important to acknowledge its limitations and the need for further examination in future studies. Firstly, while we found that employee autonomy mediates the relationship between SPL and creative service performance, other factors such as personal control may also play a role and require further investigation. Additionally, our study only considered the moderating role of proactive personality, and there may be other important factors that influence this relationship that should be explored in future studies. A possible expansion could be including voice behavior and follower traditionality.

Secondly, our study is longitudinal and relied on data collected from multiple sources. While this approach allowed us to explore the relationships between variables, this approach does not account for causality effect [95]. Therefore, future studies using experimental or quasi-experimental designs could provide stronger evidence for causation and help control for potential confounding variables.

Thirdly, our study specifically targeted Chinese employees within the information technology industry. China has distinctive cultural values, such as power distance and collectivism, which diverge from Western cultures. Thus, the generalizability of our findings to other cultural settings may be limited [96]. Moreover, companies exhibit variations in size, culture, and resources, which can significantly influence the attitudes and behaviors of both leaders and employees. The findings based on data collected from two information technology companies may also limit the generalizability of the study. Therefore, future studies should consider replicating our findings in other cultural contexts and industrial settings to enhance the external validity of our results.

### Electronic supplementary material

Below is the link to the electronic supplementary material.

Supplementary Material 1

## Data Availability

The data that support the findings of this study are available from the corresponding author, upon reasonable request.

## References

[1] Ali A, Ali SM, Xue X (2022). Motivational approach to team service performance: role of participative leadership and team-inclusive climate. J Hospitality Tourism Manage.

[2] Amabile TM (1996). Assessing the work environment for creativity. Acad Manag J.

[3] Martinaityte I, Sacramento C, Aryee S (2019). Delighting the customer: Creativity-oriented high-performance work systems, frontline employee creative performance, and customer satisfaction. J Manag.

[4] Bhattacharya S, Gupta A, Hasija S (2014). Joint product improvement by client and customer support center: the role of gain-share contracts in coordination. Inform Syst Res.

[5] Mao H (2016). Information technology resource, knowledge management capability, and competitive advantage: the moderating role of resource commitment. Int J Inf Manag.

[6] Yi H-T (2021). Examining the relationship between customer bonding, customer participation, and customer satisfaction. J Retailing Consumer Serv.

[7] Zhu H (2017). Workplace ostracism and proactive customer service performance: a conservation of resources perspective. Int J Hospitality Manage.

[8] Tariq H, Abrar M, Ahmad B. *Humility breeds creativity: the moderated mediation model of leader humility and subordinates’ creative service performance in hospitality*. Int J Contemp Hospitality Manage, 2023. Ahead of prints.

[9] Sumaneeva KA, Karadas G, Avci T (2021). Frontline hotel employees’ proactive personality, I-deals, work engagement and their effect on creative performance and proactive customer service performance. J Hum Resour Hospitality Tourism.

[10] Shin SJ, Zhou J (2007). When is educational specialization heterogeneity related to creativity in research and development teams? Transformational leadership as a moderator. J Appl Psychol.

[11] Ali A (2020). Shared leadership and team creativity: construction industry perspective. J Constr Eng Manag.

[12] Klasmeier KN, Rowold J (2020). A multilevel investigation of predictors and outcomes of shared leadership. J Organizational Behav.

[13] Pearce CL, et al. Toward a theory of meta-paradoxical leadership. Organizational Behavior and Human Decision Processes; 2019.

[14] Oldham GR, Cummings A (1996). Employee creativity: personal and contextual factors at work. Acad Manag J.

[15] Ali A, Wang H, Johnson RE (2020). Empirical analysis of shared leadership promotion and team creativity: an adaptive leadership perspective. J Organizational Behav.

[16] Zhang Y, Yang F (2021). How and when spiritual leadership enhances employee innovative behavior. Personnel Rev.

[17] Fry LW, Vitucci S, Cedillo M (2005). Spiritual leadership and army transformation: theory, measurement, and establishing a baseline. Leadersh Q.

[18] Jeon KS, Choi BK. A multidimensional analysis of spiritual leadership, affective commitment and employees’ creativity in South Korea. Leadership & Organization Development Journal; 2020.

[19] Pearce CL (2007). The future of leadership development: the importance of identity, multi-level approaches, self-leadership, physical fitness, shared leadership, networking, creativity, emotions, spirituality and on-boarding processes. Hum Resource Manage Rev.

[20] Pawar BS (2014). Leadership spiritual behaviors toward subordinates: an empirical examination of the effects of a leader’s individual spirituality and organizational spirituality. J Bus Ethics.

[21] Fry LW (2003). Toward a theory of spiritual leadership. Leadersh Q.

[22] Cacioppe R. Creating spirit at work: re-visioning organization development and leadership–part I. Leadership & Organization Development Journal; 2000.

[23] Reave L (2005). Spiritual values and practices related to leadership effectiveness. Leadersh Q.

[24] Nanus B. Visionary Leadership: creating a compelling sense of Direction for your Organization. ERIC; 1992.

[25] Conger JA, Kanungo RN. Charismatic leadership in organizations. Sage Publications; 1998.

[26] Collins RL (1996). For better or worse: the impact of upward social comparison on self-evaluations. Psychol Bull.

[27] Markow F, Klenke K. *The effects of personal meaning and calling on organizational commitment: an empirical investigation of spiritual leadership*. Int J Organizational Anal, 2005.

[28] Ryan RM (1995). Psychological needs and the facilitation of integrative processes. J Pers.

[29] Lee WR, Choi SB, Kang S-W (2021). How leaders’ positive feedback influences employees’ innovative behavior: the mediating role of voice behavior and job autonomy. Sustainability.

[30] Li H, Li F, Chen T (2018). A motivational–cognitive model of creativity and the role of autonomy. J Bus Res.

[31] Moller AC, Deci EL, Ryan RM (2006). Choice and ego-depletion: the moderating role of autonomy. Pers Soc Psychol Bull.

[32] Khoshnaw S, Alavi H. Examining the Interrelation between Job Autonomy and Job Performance: a critical literature review. Multidisciplinary Aspects of Production Engineering; 2020. p. 3.

[33] LePine MA (2016). Turning their pain to gain: charismatic leader influence on follower stress appraisal and job performance. Acad Manag J.

[34] Deci EL, Ryan RM. Cognitive evaluation theory, Intrinsic motivation and self-determination in human behavior. 1985, Springer. 43–85.

[35] Major DA, Turner JE, Fletcher TD (2006). Linking proactive personality and the big five to motivation to learn and development activity. J Appl Psychol.

[36] Chiu CC, Owens BP, Tesluk PE (2016). Initiating and utilizing shared leadership in teams: the role of leader humility, team proactive personality, and team performance capability. J Appl Psychol.

[37] Seibert SE, Crant JM, Kraimer ML (1999). Proactive personality and career success. J Appl Psychol.

[38] Gagné M, Deci EL (2005). Self-determination theory and work motivation. J Organizational Behav.

[39] Bortoluzzi G, Caporale L, Palese A (2014). Does participative leadership reduce the onset of mobbing risk among nurse working teams?. J Nurs Adm Manag.

[40] Avolio BJ (2014). E-leadership: re-examining transformations in leadership source and transmission. Leadersh Q.

[41] Shalley CE, Zhou J, Oldham GR (2004). The effects of personal and contextual characteristics on creativity: where should we go from here?. J Manag.

[42] Liu J, Chen J, Tao Y (2015). Innovation Performance in New Product Development Teams in China’s Technology Ventures: the role of behavioral integration dimensions and collective efficacy. J Prod Innov Manage.

[43] Santos CM, Uitdewilligen S, Passos AM (2015). Why is your team more creative Than Mine? The influence of Shared Mental Models on Intra-group Conflict, Team Creativity and Effectiveness. Creativity and Innovation Management.

[44] Stone DN, Deci EL, Ryan RM (2009). Beyond talk: creating autonomous motivation through self-determination theory. J Gen Manage.

[45] Deci EL, Olafsen AH, Ryan RM (2017). Self-determination theory in work organizations: the state of a science. Annual Rev Organizational Psychol Organizational Behav.

[46] Andriani S, Kesumawati N, Kristiawan M (2018). The influence of the transformational leadership and work motivation on teachers performance. Int J Sci Technol Res.

[47] Baard PP, Deci EL, Ryan RM (2004). Intrinsic need satisfaction: a motivational basis of performance and weil-being in two work settings 1. J Appl Soc Psychol.

[48] Zhang C (2019). Streams and future directions of research on work motivation based on the self-determination theory. Adv Psychol Sci.

[49] Wang M (2019). The effect of spiritual leadership on employee effectiveness: an intrinsic motivation perspective. Front Psychol.

[50] Wu W-L, Lee Y-C (2020). How spiritual leadership boosts nurses’ work engagement: the mediating roles of calling and psychological capital. Int J Environ Res Public Health.

[51] Yang F (2019). Feeling energized: a multilevel model of spiritual leadership, leader integrity, relational energy, and job performance. J Bus Ethics.

[52] Hackman JR, Oldham GR (1976). Motivation through the design of work: test of a theory. Organizational Behav Hum Perform.

[53] Cai Z (2019). How does the social context fuel the proactive fire? A multilevel review and theoretical synthesis. J Organizational Behav.

[54] Ingvaldsen JA, Rolfsen M (2012). Autonomous work groups and the challenge of inter-group coordination. Hum Relat.

[55] Shih C-T, Chen S-L, Chao M. *How autonomy-supportive leaders influence employee service performance: a multilevel study*. Serv Ind J, 2019: p. 1–22.

[56] Chen C-Y, Chen C-HV, Li C-I (2013). The influence of leader’s spiritual values of servant leadership on employee motivational autonomy and eudaemonic well-being. J Relig Health.

[57] Den Hartog DN, Belschak FD (2012). When does transformational leadership enhance employee proactive behavior? The role of autonomy and role breadth self-efficacy. J Appl Psychol.

[58] Pattnaik SC, Sahoo R. Employee engagement, creativity and task performance: role of perceived workplace autonomy. South Asian Journal of Business Studies; 2020.

[59] Robert LP, You S (2018). Are you satisfied yet? Shared leadership, individual trust, autonomy, and satisfaction in virtual teams. J Association Inform Sci Technol.

[60] Volmer J, Spurk D, Niessen C (2012). Leader–member exchange (LMX), job autonomy, and creative work involvement. Leadersh Q.

[61] Brown DJ (2007). Antecedents and consequences of the frequency of upward and downward social comparisons at work. Organ Behav Hum Decis Process.

[62] DeRue DS (2011). Adaptive leadership theory: leading and following as a complex adaptive process. Res Organizational Behav.

[63] Tornau K, Frese M (2013). Construct clean-up in proactivity research: a meta‐analysis on the nomological net of work‐related proactivity concepts and their incremental validities. Appl Psychol.

[64] Grant AM, Patil SV (2012). Challenging the norm of self-interest: minority influence and transitions to helping norms in work units. Acad Manage Rev.

[65] Bateman TS, Crant JM (1993). The proactive component of organizational behavior: a measure and correlates. J Organizational Behav.

[66] Bahadur W, Ali A. Linking leader humility with service performance: the role of service climate and customer mistreatment. Asian Business & Management (Forthcoming); 2021.

[67] Schilpzand P, Houston L, Cho J (2018). Not too tired to be proactive: Daily empowering leadership spurs next-morning employee proactivity as moderated by nightly sleep quality. Acad Manag J.

[68] Liu W (2019). New directions for exploring the consequences of proactive behaviors: introduction to the special issue. J Organizational Behav.

[69] Ye Y (2019). Family ostracism and proactive customer service performance: an explanation from conservation of resources theory. Asia Pac J Manage.

[70] Hayes AF (2015). An index and test of linear moderated mediation. Multivar Behav Res.

[71] Preacher KJ, Rucker DD, Hayes AF (2007). Addressing moderated mediation hypotheses: theory, methods, and prescriptions. Multivar Behav Res.

[72] Podsakoff PM (2003). Common method biases in behavioral research: a critical review of the literature and recommended remedies. J Appl Psychol.

[73] Ali A, Wang H, Boekhorst JA (2023). A moderated mediation examination of shared leadership and team creativity: a social information processing perspective. Asia Pac J Manage.

[74] Shin SJ, Zhou J (2003). Transformational leadership, conservation, and creativity: evidence from Korea. Acad Manag J.

[75] Brislin RW. *Translation and content analysis of oral and written materials. In H.C. Triandis & J.W. Berry, editors, Handbook of crosscultural psychology: Vol. 2. Methodology (pp. 389–444)*. 1980, Boston: Allyn & Bacon.

[76] Yang F, Huang X, Wu L (2019). Experiencing meaningfulness climate in teams: how spiritual leadership enhances team effectiveness when facing uncertain tasks. Hum Resour Manag.

[77] Pham NT et al. *Common good human resource management, ethical employee behaviors, and organizational citizenship behaviors toward the individual*. Hum Resource Manage J, 2023. Ahead of prints.

[78] Srivastava S (2022). Happiness at work through spiritual leadership: a self-determination perspective.

[79] Beehr TA (1976). Perceived situational moderators of the relationship between subjective role ambiguity and role strain. J Appl Psychol.

[80] Wang G, Netemeyer RG (2004). Salesperson creative performance: conceptualization, measurement, and nomological validity. J Bus Res.

[81] Ali A (2020). Promoting Shared Leadership to Improve Team Innovation: an adaptive structuration theory perspective. Acad Manag Proc.

[82] Cao X, Ali A (2018). Enhancing team creative performance through social media and transactive memory system. Int J Inf Manag.

[83] He W (2020). Different roles of shared and vertical leadership in promoting team creativity: cultivating and synthesizing team members’ individual creativity. Pers Psychol.

[84] Cao X (2021). A socio-technical system approach to knowledge creation and team performance: evidence from China. Inform Technol People.

[85] Tang Y, et al. Comparisons draw us close: the influence of leader-member exchange dyadic comparison on coworker exchange. Personnel Psychology; 2021.

[86] Tariq H, Ding D (2018). Why am I still doing this job? The examination of family motivation on employees’ work behaviors under abusive supervision. Personnel Rev.

[87] Eissa G, Lester SW (2017). Supervisor role overload and frustration as antecedents of abusive supervision: the moderating role of supervisor personality. J Organizational Behav.

[88] Hu Lt, Bentler PM (1999). Cutoff criteria for fit indexes in covariance structure analysis: conventional criteria versus new alternatives. Struct Equation Modeling: Multidisciplinary J.

[89] Podsakoff PM, Organ DW (1986). Self-reports in Organizational Research: problems and prospects. J Manag.

[90] Brockner J (1997). When Trust matters: the moderating effect of Outcome favorability. Adm Sci Q.

[91] Harman HH. Modern factor analysis. University of Chicago Press; 1976.

[92] Bayighomog SW, Araslı H (2019). Workplace spirituality – customer engagement Nexus: the mediated role of spiritual leadership on customer–oriented boundary–spanning behaviors. Serv Ind J.

[93] Egel E, Fry LW. *Impact of Spiritual Leadership on Team Creativity*. in *Academy of Management Proceedings*. 2015. Academy of Management Briarcliff Manor, NY 10510.

[94] Saeed I (2022). Towards examining the link between workplace spirituality and workforce agility: exploring higher educational institutions. Psychol Res Behav Manage.

[95] Kundro TG. *The benefits and burdens of work moralization on creativity* Academy of Management Journal, 2022. In press.

[96] Hobfoll SE (2001). The influence of culture, community, and the nested-self in the stress process: advancing conservation of resources theory. Appl Psychol.

