# Supplementary material for: Spiritual leadership influence on employee creative service performance: a moderated mediation analysis
Source: BMC Psychol. 2023 Sep 4;11:262. doi: 10.1186/s40359-023-01294-0 (PMC10478217; doi:10.1186/s40359-023-01294-0)
Supplement: Supplementary file 1 — Supplementary Material 1 [file 40359_2023_1294_MOESM1_ESM.docx]

**Spiritual leadership (**[**Pawar, 2014**](#_ENREF_2)**)**

1. Through his/her behaviors toward subordinates, my leader expresses respect for subordinates’ values.
2. Through his/her behaviors toward subordinates, my leader expresses that his/her cares for his subordinates.
3. Through his/her behaviors toward subordinates, my leader expresses my leader concern for subordinates.
4. My leader carefully listens to subordinates.
5. My leader recognizes subordinates’ contributions to workplace.
6. My leader deals with subordinates in a fair manner.
7. My leader behavior toward subordinates reflects ethical/moral values
8. My leader is honest in dealing with subordinates
9. My leader behavior toward subordinates reflects his/her awareness that he/she is just an ordinary human being.

**Employee autonomy (**[**Beehr, 1976**](#_ENREF_1)**)**

1. I have a lot of say over what happens on my job.
2. I have enough authority to do my best.
3. My job allows me to make a lot of decisions on my own.
4. I have enough freedom as to how I do my work.

**Proactive personality (**[**Seibert et al., 1999**](#_ENREF_3)**)**

1. I am constantly on the lookout for new ways to improve my life.
2. Wherever I have been, I have been a powerful force for constructive change.
3. Nothing is more exciting than seeing my ideas turn into reality.
4. If I see something I don't like, I fix it.
5. No matter what the odds, if I believe in something I will make it happen.
6. I love being a champion for my ideas, even against others' opposition.
7. I excel at identifying opportunities.
8. I am always looking for better ways to do things.
9. If I believe in an idea, no obstacle will prevent me from making it happen.
10. I can spot a good opportunity long before others can.

**Employee creative service performance (**[**Wang & Netemeyer, 2004**](#_ENREF_4)**)**

1. This employee carries out service tasks in ways that are resourceful.
2. This employee comes up with new ideas for satisfying customer needs.
3. This employee generates and evaluates multiple alternatives for novel customer problems.
4. This employee has fresh perspectives on old problems.
5. This employee improvises methods for solving a problem when an answer is not apparent.
6. This employee generates creative ideas for service delivery.

Beehr, T. A. (1976). Perceived situational moderators of the relationship between subjective role ambiguity and role strain. *Journal of Applied Psychology*, *61*, 35-40. https://doi.org/10.1037/0021-9010.61.1.35

Pawar, B. S. (2014). Leadership spiritual behaviors toward subordinates: An empirical examination of the effects of a leader’s individual spirituality and organizational spirituality. *Journal of Business Ethics*, *122*(3), 439-452. https://doi.org/https://doi.org/10.1007/s10551-013-1772-5

Seibert, S. E., Crant, J. M., & Kraimer, M. L. (1999). Proactive personality and career success. *Journal of Applied Psychology*, *84*(3), 416-427. https://doi.org/10.1037/0021-9010.84.3.416

Wang, G., & Netemeyer, R. G. (2004). Salesperson creative performance: conceptualization, measurement, and nomological validity. *Journal of Business Research*, *57*(8), 805-812. https://doi.org/https://doi.org/10.1016/S0148-2963(02)00483-6
